# Role of Individual Amino Acid Residues Directly Involved in Damage Recognition in Active Demethylation by ABH2 Dioxygenase

**DOI:** 10.3390/ijms26146912

**Published:** 2025-07-18

**Authors:** Anastasiia T. Davletgildeeva, Timofey E. Tyugashev, Mingxing Zhao, Alexander A. Ishchenko, Murat Saparbaev, Nikita A. Kuznetsov

**Affiliations:** 1 Institute of Chemical Biology and Fundamental Medicine, Siberian Branch of Russian Academy of Sciences, 630090 Novosibirsk, Russia; tyugashev@niboch.nsc.ru; 2 Department of Natural Sciences, Novosibirsk State University, 630090 Novosibirsk, Russia; hdzhaomingxing@163.com; 3 Groupe Mechanisms of DNA Repair and Carcinogenesis, CNRS UMR9019, Gustave Roussy Cancer Campus, Université Paris-Saclay, CEDEX, F-94805 Villejuif, France; alexander.ishchenko@gustaveroussy.fr (A.A.I.); murat.saparbaev@gustaveroussy.fr (M.S.)

**Keywords:** DNA repair, DNA dioxygenase ABH2, DNA methylation, conformational dynamics, MD modeling, functional role of amino acid residues

## Abstract

The enzyme ABH2, one of nine human DNA dioxygenases of the AlkB family, belongs to the superfamily of Fe(II)/α-ketoglutarate-dependent dioxygenases and plays a crucial role in the direct reversal repair of nonbulky alkyl lesions in DNA nucleobases. ABH2 has broad substrate specificity, directly oxidizing DNA damages such as *N*^1^-methyladenine, *N*^3^-methylcytosine, 1,*N*^6^-ethenoadenine, 3,*N*^4^-ethenocytosine, and a number of others. In our investigation, we sought to uncover the subtleties of the mechanisms governing substrate specificity in ABH2 by focusing on several critical amino acid residues situated in its active site. To gain insight into the function of this enzyme, we performed a functional mapping of its active site region, concentrating on pivotal residues, participating in forming a damaged binding pocket of the enzyme (Val99 and Ser125), as well as the residues directly involved in interactions with damaged bases, namely Arg110, Phe124, Arg172, and Glu175. To support our experimental data, we conducted a series of molecular dynamics simulations, exploring the interactions between the ABH2 mutant forms, bearing corresponding substitutions and DNA substrates, and harboring various types of methylated bases, specifically *N*^1^-methyladenine or *N*^3^-methylcytosine. The comparative studies revealed compelling data indicating that alterations in most of the studied amino acid residues significantly influence both the binding affinity of the enzyme for DNA and its catalytic efficiency. Intriguingly, the findings suggest that the mutations impact the catalytic activity of ABH2 to a greater extent than its ability to associate with DNA strands. Collectively, these results show how changes to the active site affect molecular dynamics and reaction kinetics, improving our understanding of the substrate recognition process in this pivotal enzyme.

## 1. Introduction

The alkylation of DNA can be caused by both endogenous and exogenous sources, and this can lead to cytotoxicity and the development of cancer-related mutations [1,2,3,4]. Accordingly, there are several pathways to repair the alkylated damages in DNA through its repair, namely by the activity of the DNA glycosylases, O^6^-methylguanine DNA methyltransferases, and AlkB-type dioxygenases [2,3,5,6,7]. The AlkB family dioxygenases belong to a broad family of α-ketoglutarate (αKG) and non-heme Fe(II)-dependent oxygenases [8,9,10]. AlkB homologs are common in eukaryotic cells and are found in most bacteria. These enzymes are involved in the demethylation of a wide range of substrates, including DNA, RNA, and proteins [5,11,12]. To date, nine AlkB homologs have been identified in mammals, including ABH1 to ABH8 and FTO [13,14,15,16]. All of these enzymes possess a functional dioxygenase domain in their structure. However, they are localized in different regions of the cell, catalyze the dealkylation of different substrates, and therefore, have different biological significance [17].

Thus, AlkB and ABH3 preferably repair methylated damages in single-stranded nucleic acids (ssDNA), while ABH2 is more efficient in repairing double-stranded DNA (dsDNA) [5,18,19,20]. The efficiency of DNA repair with the participation of these enzymes also depends on the specific type of nucleic acid base and on the nature and position of the alkylated group. A number of monoalkylated DNA bases, namely *N*^1^-methyladenine (m^1^A), *N*^3^-methylcytosine (m^3^C), *N*^2^-methylguanine (m^2^G), *N*^6^-methyladenine (m^6^A), and *N*^4^-methylcytosine (m^4^C), are known to be among the substrates of the members of the AlkB dioxygenases family. The enzymes of this family can also repair some exocyclic lesions such as 1,*N*^6^-ethenoadenine (εA), 3,*N*^4^-ethenocytosine (εC), and *N*^2,3^-ethenoguanine (εG) [5,21,22].

The interpretation of the crystal structures of AlkB and homologs has shown that all the enzymes with the resolved structure have a common modified double-stranded β-helix (DSBH) core fold, which consists of eight β-strands surrounding the active site of the enzyme [18,19,23]. While the prokaryotic enzyme AlkB forms contacts almost exclusively with the damaged DNA strand and uses the mechanism of flipping out of the damaged base into the active site of the enzyme to carry out the catalytic reaction, ABH2 has a specific hydrophobic hairpin containing Phe102, which intercalates into the duplex, filling the space vacated by the everted base [18,19]. ABH2 also has additional DNA-binding motifs that span the complementary dsDNA strand. The enzyme binds the complementary intact DNA strand using a positively charged RKK loop (Arg241, Lys242, and Lys243) and a long flexible loop containing the DNA-binding residues Arg198, Gly204, and Lys205 [18,19,20]. The currently known data indicate that three amino acid residues are used to coordinate the Fe(II) ion at the active site of the enzyme dioxygenases from the AlkB family—two His and one Asp. For ABH2, it is an amino acid triad, His171, Asp173, and His236 [24].

Beginning the work dedicated to the elucidation of the role of amino acid residues of the ABH2 enzyme in the catalytic process, our group performed the analysis of available structural data, which allowed us to compile a list of the amino acid residues that may be of interest because of their location near the active site and the damaged-base-binding pocket [25]. Back then, we identified the Val99, Arg110, Ser125, and Ile168 residues as forming the wall of the damaged-base pocket. The Tyr122, Phe124, His171, Asp173, and Glu175 residues were considered to come into direct contact with the damaged bases.

Over the past couple of decades, several studies have been published examining the role of key amino acid residues of the ABH2 enzyme, including our previous paper [25,26,27,28]. Thus, Lee and colleagues showed that replacing Asp173 with Ala inactivates the enzyme, while H236A only leads to a slight decrease in activity towards both m^1^A and m^3^C bases. Our previous work also demonstrated that the D173A substitution significantly reduced ABH2 activity against both lesions. A similar effect was observed for the mutant form containing the Y122A substitution. The Arg110 substitution also led to a complete loss of enzymatic activity, while the ABH2 I168A mutant form, also containing a substitution in the wall of the damaged-base pocket, had reduced activity only towards m^3^C-containing DNA. It was found that the intercalating loop residues Phe102 and Val101 appear to have different degrees of importance for the repair of ss and dsDNA. At the same time, neighboring Val99, participating in a hydrophobic network of the enzyme, was also demonstrated to be important for demethylase activity toward ss and dsDNA substrates. Glu175 and Phe124 have also been shown to play a role in the specific selection and stabilization of nucleotide bases in the active site for repair.

In the current study, we present a mutational analysis of six amino acid residues located at the area of the active site of human DNA dioxygenase ABH2 and potentially involved in substrate binding, substrate recognition, and enzyme catalytic activity. The amino acids Val99 and Ser125 were chosen as forming the wall of the damaged-base binding pocket, and the Arg110, Phe124, Arg172, and Glu175 were chosen based on their ability to directly interact with the methylated base lesions from distinct spatial points. By focusing on these six residues, we aimed to understand their individual contributions to the recognition process and the overall catalytic function of ABH2. To investigate the role of these amino acid residues, we created single-point mutant forms by substituting the selected amino acids with alanine. By combining computational molecular dynamics simulations with experimental assessments of DNA binding and enzyme activity, we were able to unravel the contribution of these selected residues to ABH2 functions.

## 2. Results and Discussion

### 2.1. Choosing Potentially Important Amino Acid Residues Forming Specific Contacts with Damaged Bases

**Figure 1:**
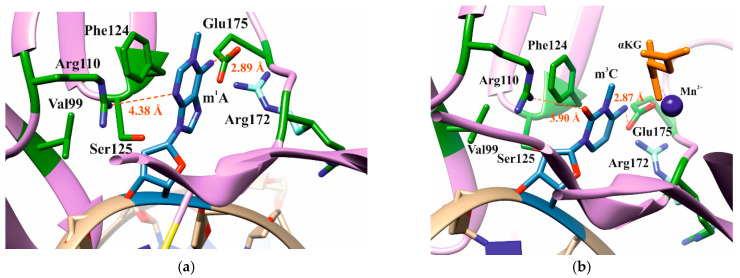
Residues substituted by site-directed mutagenesis in the active site area of ABH2 that is in complex with m^1^A-containing DNA ((**a**), PDB ID: 3BTY) or m^3^C-containing DNA ((**b**), PDB ID: 3RZJ).

An analysis of the structure of the ABH2 enzyme bound to m^3^C-containing [PDB ID: 3RZJ] or m^1^A-containing DNA [PDB ID: 3BTY] allowed us to pick a number of amino acid residues that were of potential interest for studying their role in the step-by-step damaged-base recognition process. This analysis of the structures of ABH2–DNA complexes (Figure 1) revealed that amino acid residues Val99, Arg110, and Ser125 participate in the formation of the wall of the damaged-base pocket. Also, Arg110, as well as Glu175, is engaged in contact with both m^3^C and m^1^A (damaged bases) placed inside the active site. This observation suggests the possible importance of these residues for the damage-checking mechanism of substrate specificity. Phe124 is able to stack with all types of flipped-out damaged bases at the active side of the enzyme. As for the Arg172 residue, based on the analysis of the rotamer variants, it was hypothesized that this amino acid residue is able to turn toward the damaged base. Therefore, these amino acid residues were chosen to verify their participation in DNA binding, catalytic complex formation, and catalysis under steady-state conditions.

### 2.2. MD Simulations of Mutant Forms of the Enzyme

The functions of the amino acid residues Val99, Arg110, Phe124, Ser125, Arg172, and Glu175 in recognition of the methylated bases were estimated by MD simulations. Previous modeling studies of the AlkB family proteins have used both a point charge model approach, in which the non-heme iron atom in the active site of AlkB family dioxygenases was represented by a +2 charge with 12-6 Lennard-Jones parameters [29], and a bonded model approach, more suitable for transition metals [24,30,31,32,33], though less able to account for active site alteration when comparing enzyme variants. Therefore, in the present work, we employed a nonbonded cationic dummy model [31], enabling the comparative modeling of various mutant forms while preserving the coordination geometry of the iron ion.

ABH2 cross-linked with DNA in the presence of the Mn^2+^ ion and cofactor αKG (PDB ID: 3RZJ for m^3^C-containing DNA and PDB ID: 3BUC for m^1^A-containing DNA) were used as initial simulation structures [18,19]. Crystal structures for crystallization-modified ABH2 WT complexes with DNA substrates containing m^3^C or m^1^A do not display any notable differences in amino acid interactions within the active site. Similarly, molecular dynamics show little difference between the complexes of ABH2 with DNA substrates containing m^3^C or m^1^A, both for the WT protein as well as for the mutant forms.

**Figure 2:**
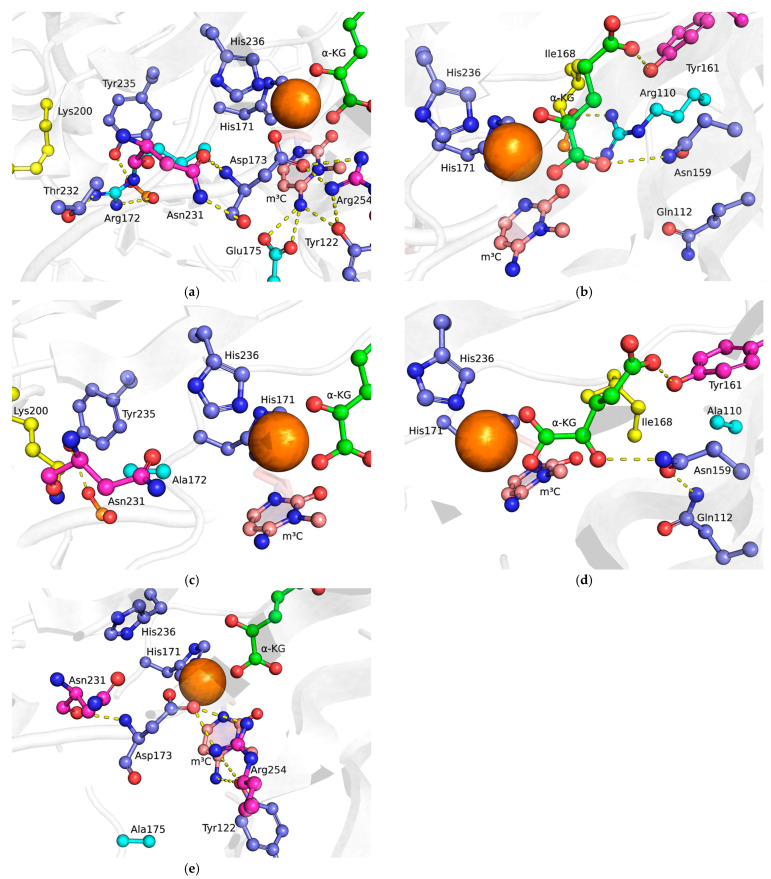
Interaction of amino acid residues in the active site of ABH2 WT and its mutant forms in complex with DNA substrate containing m^3^C: (**a**,**b**) ABH2 WT; (**c**) ALKBH2 R172A; (**d**) ALKBH2 R110A; (**e**) ALKBH2 E175A. Key residues are shown as sticks: mutated residues are colored cyan, residues that shift in mutant forms are yellow, some of the residues that form hydrogen bonds are magenta, and the rest are violet. Hydrogen bonds are shown as dashed yellow lines; Fe(II) ion—as the orange sphere.

**Figure 3:**
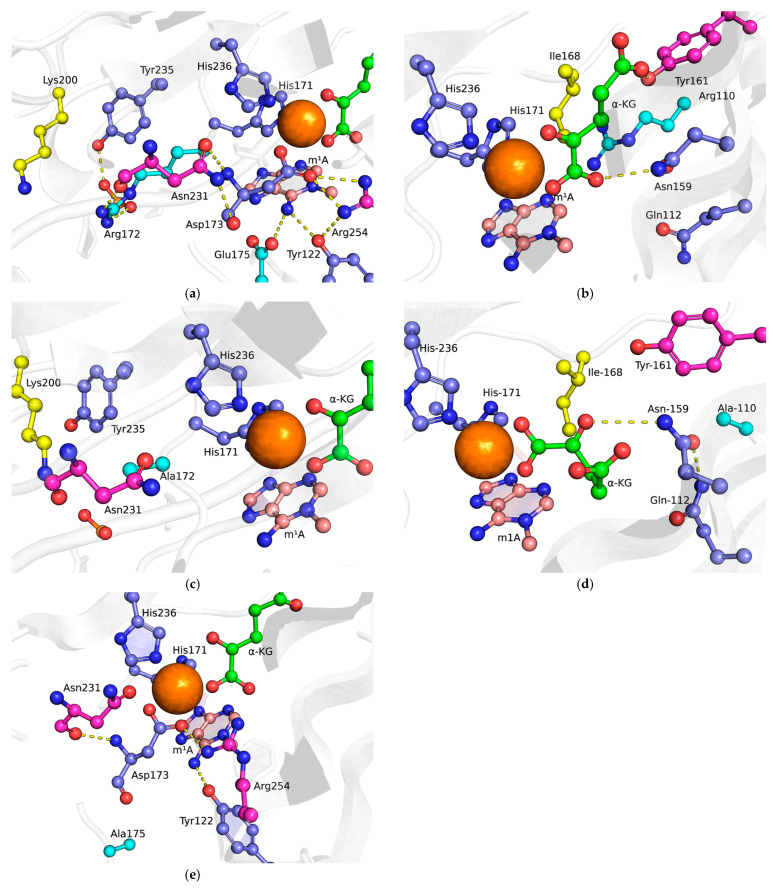
Interaction of amino acid residues in the active site of ABH2 WT and its mutant forms in complex with DNA substrate containing m^1^A: (**a**,**b**) ABH2 WT; (**c**) ALKBH2 R172A; (**d**) ALKBH2 R110A; (**e**) ALKBH2 E175A. Key residues are shown as sticks: mutated residues are colored cyan, residues that shift in mutant forms are yellow, some of the residues that form hydrogen bonds are magenta, and the rest are violet. Hydrogen bonds are shown as dashed yellow lines; Fe(II) ion—the orange sphere.

In the structure of the ABH2 WT complex with DNA substrate containing m^3^C (Figure 2a,b) or m^1^A (Figure 3a,b) lesions, residues Glu175 and Tyr122 participate in the damaged base’s recognition by forming hydrogen bonds with its amino group [19]. Additionally, residues Glu175, Tyr122, and Arg254 and the amino group of m^3^C form a network of hydrogen bonds that holds residue Asp173, which is monodentately coordinated to Fe^2+^ ion. Arg110 forms a salt bridge with a phosphate group of lesioned DNA strand, thus helping to stabilize the damaged base in the active site. Arg110 also forms a hydrogen bond with the amide group of Asn159, which, in turn, forms hydrogen bonds with the α-carboxyl group of αKG (on a part of the trajectory) and the peptide backbone of Asn250. Also, as part of the trajectory, Gln112 forms a hydrogen bond with Asn159. Tyr161 forms a hydrogen bond with the O atom of the γ-carboxyl group of αKG. The Arg172 side chain forms a salt bridge with a phosphate group of the lesioned DNA strand, and hydrogen bonds with the O atom of the Asn231 peptide backbone and the hydroxyl group of Thr232.

In our previous work (publication in process), we modeled complexes of ABH2 mutant forms, bearing V99A, F124A, or S125A amino acid substitutions, with DNA containing m^1^A or m^3^C to analyze conformational rearrangements induced by the selected amino acid substitution. Briefly, the obtained MD model of ABH2 V99A revealed that this substitution leads to changes in the mobility of the L2 loop (comprising Tyr122, Phe124, and Ser125), participating in nucleotide recognition [34]. This, along with the slight changes in the L1 intercalating loop positioning (V101, F102, and G103 amino acids) and αKG orientation in a suboptimal position for catalysis due to the shortening of the distance between Gln112 and Asn159, indicates that the V99A substitution most likely should affect the catalytic efficacy of the ABH2 enzyme.

The substitution of F124A led to frequent changes in the binding type of the Fe^2+^ ion and αKG. The introduction of Ala124 also resulted in the distancing of the L2 loop from the DNA substrate, mainly by changing the position of Ser125. The F124A substitution also influenced the interaction of the hydroxyl group of the side chain of Tyr161 residue and the amino group of the side chain of Arg248 with αKG. In general, all these noticeable effects calculated for F124A indicate the important role of this amino acid residue in the catalytic process carried out by ABH2. The S125A substitution also affects the L2 loop, but these changes were only noticed for the complex with m^1^A-containing DNA substrate.

In the obtained MD model of the complex of ABH2 R110A with the m^3^C DNA substrate (Figure 2b), the Arg110 side chain interaction with the DNA is lost due to its substitution with alanine. As a consequence, the side chains of Ile168 and Gln112 shift into the space previously occupied by the Arg110 side chain in the ABH2 WT complex. For Ile168, this movement increases the distance between its δ-C atom and ε-N atom of His171. This rearrangement of the side chain hydrogen bond network results in an altered binding mode of the αKG, with the Asn159 amide group forming hydrogen bonds with oxygen atoms of carbonyl and γ-carboxyl groups of αKG, while the hydrogen bond between the Tyr161 hydroxyl group and the γ-carboxyl group is weakened compared to the wild-type complex. Within the constraints of the model system employed, these structural changes suggest that the R110A mutant adopts catalytically unfavorable active site conformation distinct from the wild-type protein.

The R172A substitution (Figure 2c) results in the loss of a hydrogen bond with the Asn231 residue. Instead, the Lys200 residue side chain rotates and shifts into the space previously occupied by the side chain of Arg172 in the wild-type enzyme, forming a new hydrogen bond with the peptide backbone of Asn231, and bringing it closer to the sugar-phosphate backbone of the DNA. In the wild-type enzyme–DNA complex, the amino group of Lys200 was positioned farther from the phosphate group of the nucleotide flanking the damaged base at the 5′-end, whereas in the mutant enzyme, this distance was significantly reduced. Additionally, Lys200 forms a hydrogen bond with the peptide backbone of Asn231, an interaction that was absent or much weaker in the wild-type enzyme. Thus, unlike the R110A substitution, the replacement of Arg172 with Ala172 does not affect the stabilization of the damaged base in the active site or the coordination of αKG and Asp173 with the Fe^2+^ ion.

The E175A substitution (Figure 2d) disrupts the hydrogen bond network formed by residues Asp173, Glu175, Tyr122, Asn231, and Arg254 and the amino group of the everted methylated nucleobase. In the model, this is expressed as altered Fe^2+^ ion coordination, with the Asp173 side chain forming a bidentate interaction with the Fe^2+^ ion, displacing both αKG and the Fe-coordinating His236 side chain. Additionally, the hydrogen bond between the Asn213 side chain and the Asp173 backbone shifts to a hydrogen bond between the Asn231 backbone and the Asp173 side chain. Overall, the active site structure is severely disrupted, providing a structural explanation for the previously noted complete loss of activity in this mutant [26].

### 2.3. Circular Dichroism (CD) Spectra

**Figure 4:**
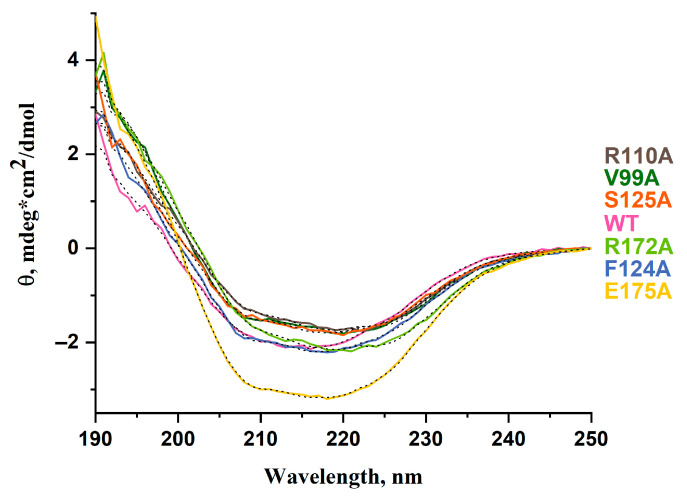
CD spectra of WT ABH2 (pink) and of mutant forms featuring the V99A (dark green), R110A (gray), F124A (blue), S125A (orange), R172A (green), or E175A (yellow) substitutions. The results of fitting are shown as black dotted lines.

To evaluate the impact of the Val99, Arg110, Phe124, Ser125, Arg172, or Glu175 amino acids on the activity of the ABH2 enzyme toward DNA containing m^3^C or m^1^A, the ABH2 mutant forms featuring the substitutions of these amino acids to alanine were purified. To estimate the effects of the chosen substitutions on the global structure of the enzyme, the CD spectra of these recombinant proteins were recorded (Figure 4). The results showed similar structural patterns for the ABH2 WT and most of its mutant forms, indicating that these substitutions do not cause a global alteration of the secondary structure of the protein. This suggests that, despite the MD results, which clearly indicate local changes in the structure of the studied mutant forms of ABH2, the CD spectra analysis approach does not allow reflecting them and can mainly be used to assess global changes in the overall protein structure. Nevertheless, the CD spectra of the R172A and E175A mutant forms had a slight displacement of local minima compared to the WT spectra. CD spectra obtained for the ABH2 WT enzyme exhibit two pronounced local minima at 208 and 219 nm. In the case of R172A, the first minimum moves to 211 nm and the second minimum moves to 224 nm. On the contrary, for the CD spectra characterizing the E175A mutant form, the second minimum is displaced to 218 nm, and the overall spectrum has more pronounced amplitude compared to all other spectra. These differences could indicate the complex impact on the overall structure of the ABH2 mutant forms featuring R172A or E175A substitutions.

The results from Table 1, as calculated by means of the web resource for the analysis of the CD spectra, indicated that β-sheets were the main ordered secondary structures of ABH2 WT (34%). As for the mutant forms, this type of structure remained prevailing for them as well. Yet the percentage of the β-sheets changed significantly for the E175A mutant form in absolute values (up to 28%), although it should be noted that due to the limitations of the method, these values of the β-sheets content still lay within the margin of error, except that the difference between the ABH2 WT and its mutant forms was within 2% in absolute values. The results of the fitting of the CD spectra of the ABH2 E175A mutant form also showed a 5% increase in the percentage of the α-helixes structures. At the same time, the R110A and S125A mutant forms demonstrated 5 and 4% decreases in the percentage of the α-helixes structures in absolute values, although these values stayed within the margin of error. Considering the fact that the calculated amount of the β-turn structures remained within 11–12% diapason for all the enzymes studied, it could be concluded that the differences in the CD spectra between ABH2 WT and the R110A, S125A, and E175A mutant forms are mostly conditioned by the redistribution in the relative amounts of the α-helixes and β-sheets.

### 2.4. The Melting Temperature

**Figure 5:**
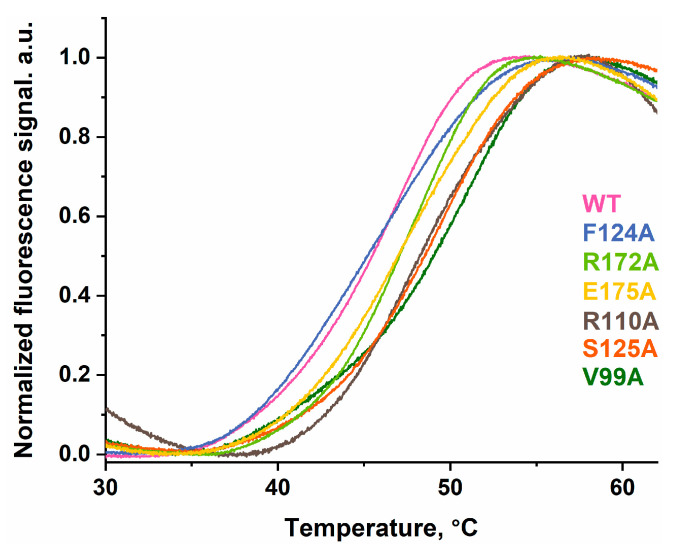
Representative thermal melt curves for WT ABH2 (pink) and its mutant forms featuring the V99A (dark green), R110A (gray), F124A (blue), S125A (orange), R172A (green), or E175A (yellow) substitutions.

To estimate the effects of the Val99, Arg110, Phe124, Ser125, Arg172, or Glu175 amino acid substitutions on the thermal stability of the ABH2 enzyme, the melting temperature was calculated using the thermal shift assay (Figure 5).

Measuring the thermal stability of the enzymes revealed that the T_m_ value lies in a range between 46 and 50.4 °C for the tested variants, with the value for ABH2 WT being 46.7 °C (Table 2). It is interesting that the difference between the lowest T_m_ value for the F124A variant and the WT enzyme is less than 1 °C and stays within the measurement error range. As for the other mutations, all of them led to an increase in the T_m_ value up to a 3.7 °C difference with the WT for the V99A variant. This may indicate that the replacement of some amino acid residues with alanine increases the thermal stability of the ABH2 enzyme, especially Val99, Arg110, and Ser125. It is interesting that two of three of these mutant forms featuring the substitutions of these amino acids (R110A and S125A) were also characterized by a sufficient decline in the percentage of the α-helixes structures according to CD spectra fitting (Table 1). This suggests that approaches to analyzing CD spectra and estimating the melting temperature of a protein globule can yield results that reflect different characteristics of the proteins analyzed. Amino acid substitutions that change the percentage of structural elements such as α-helices can introduce local changes in the structure of this nature, due to interactions with neighboring amino acid residues. Such local changes cannot be detected by CD spectral analysis, but nevertheless may have an effect on the thermal stability of enzyme domains and the functionality of proteins.

### 2.5. Equilibrium Binding of ABH2 Mutant Forms to Methylated DNA

**Figure 6:**
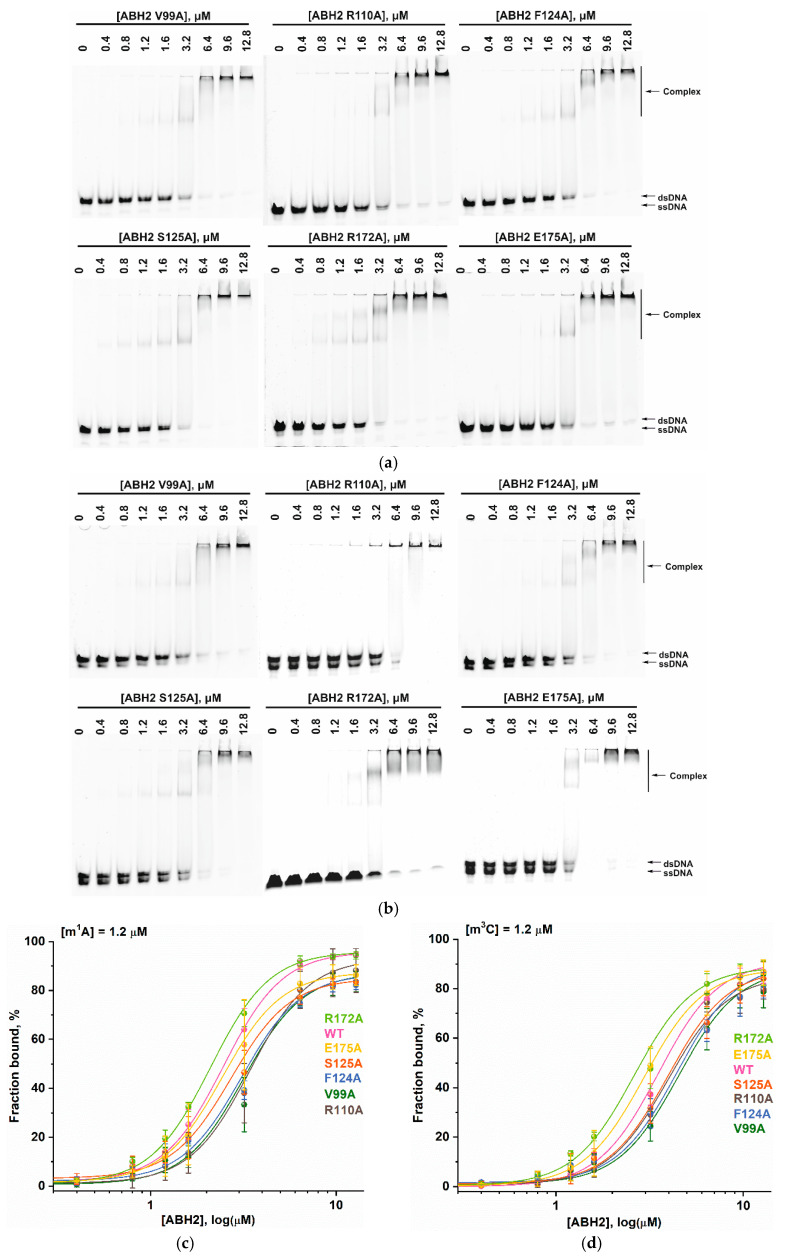
The EMSA of the ABH2 mutant forms V99A, R110A, F124A, S125A, R172A, and E175A. (**a**) EMSA of V99A, R110A, F124A, S125A, R172A, and E175A mutant forms with 1.2 µM of FAM-labeled m^1^A-containing DNA. (**b**) EMSA of V99A, R110A, F124A, S125A, R172A, and E175A mutant forms with 1.2 µM of FAM-labeled m^3^C-containing DNA. (**c**) Fractions of bound FAM-labeled m^1^A-containing DNA plotted against ABH2 concentrations, with curve fitting of the obtained data. (**d**) The fractions of bound FAM-labeled m^3^C-containing DNA plotted against ABH2 concentrations, with curve fitting of the obtained data. In each panel, the concentration of DNA is 1.2 µM, and concentrations of the enzyme are 0, 0.4, 0.8, 1.2, 1.6, 3.2, 6.4, 9.6, and 12.8 µM. The data for ABH2 WT were obtained earlier [25] and are demonstrated here for comparison.

**Figure 7:**
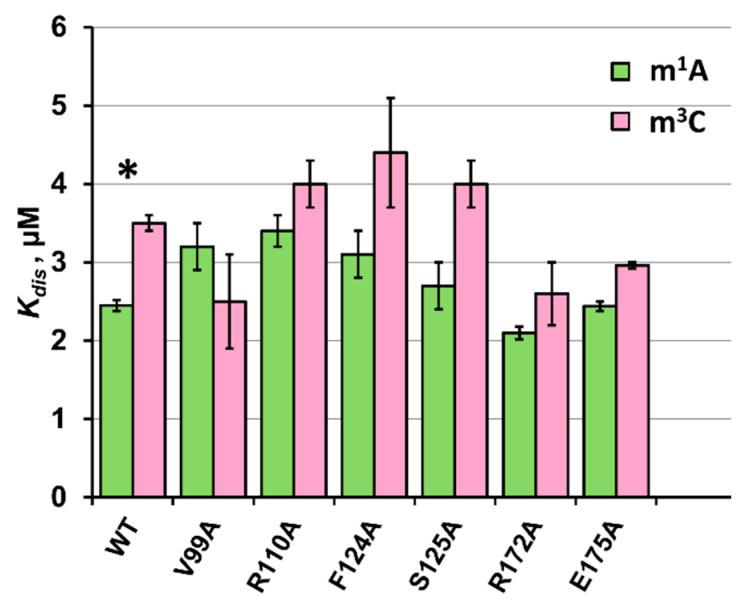
Analysis of binding affinity of ABH2 mutant forms V99A, R110A, F124A, S125A, R172A, and E175A in an EMSA. Dissociation constants (*K_d_*) were determined by the fitting of the experimental data points to the Hill equation (Equation (1)). Error bars represent the standard deviation of three technical replicates. * *K_d_* characterizing binding affinity of the ABH2 WT was obtained earlier [25] and is demonstrated here for comparison.

To establish the impact of the studied amino acids on the binding abilities of the ABH2 enzyme, electrophoretic mobility shift assays (EMSAs) of the V99A, R110A, F124A, S125A, R172A, and E175A mutant forms were performed in the presence of 5′-FAM-labeled oligonucleotides containing m^1^A or m^3^C as a lesion (Figure 6a,b). The enzymes were titrated with 17 nt methylated dsDNA. Equilibrium dissociation constants (*K_d_*) for complexes of the enzyme with target dsDNA (Figure 6c,d) were calculated by means of Equation (1). The obtained dissociation constants were in a range of 2.1–3.4 µM for the binding to the m^1^A-containing DNA and 2.5–4.4 µM for the binding to the m^3^C-containing DNA (Figure 7).

The obtained results of the binding assay indicate that the amino acid substitutions under study do not bring significant changes to the affinity of the ABH2 enzyme to dsDNA containing methylated bases m^1^A or m^3^C. The stability of the complex with m^1^A-containing dsDNA is slightly higher compared to the m^3^C-containing DNA, with an exception in the case of the V99A mutant form. The range of *K_d_* values for m^1^A-containing DNA binding was the following: R172A < E175A = WT < S125A < F124A < V99A < R110A. As for the m^3^C-containing dsDNA, the range of *K_d_* values was similar, except for the fact that the *K_d_* values for R110A and V99A mutant forms changed their position in a range, and for the V99A, this change was significant: V99A < R172A < E175A < WT < S125A = R110A < F124A. The greatest *K_d_* value for the binding of the m^1^A-containing dsDNA was observed in the case of R110A, and it was only 1.6-fold greater than the smallest one (in the case of R172A). As for the m^3^C-containing dsDNA, the difference between the highest and the lowest *K_d_* values was 1.9 µM. These results mean that all of the introduced amino acid substitutions only have a modest influence on the processes involving the binding of a methylated DNA substrate. In general, the tendency is as follows: the substitution of Arg110, Phe124, or Ser125 weakens the binding affinity of the ABH2 enzyme both in the cases of m^1^A- and m^3^C-containing dsDNA. It is interesting that the R172A and E175A substitutions, on the contrary, either do not have a significant impact on the binding affinity of the ABH2 enzyme (as in the case of E175A and m^1^A-containing DNA) or seem to improve it. The most peculiar case is the Val99 substitution, which leads to a decrease in the binding affinity of the ABH2 enzyme to the m^1^A-containing dsDNA, simultaneously increasing it toward m^3^C-containing dsDNA.

### 2.6. Activity of ABH2 Mutant Forms Toward DNA Containing m^1^A or m^3^C

**Figure 8:**
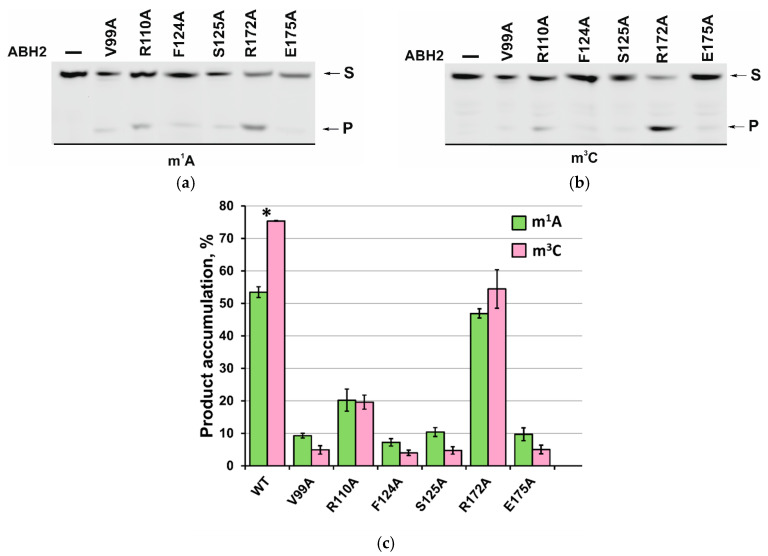
PAGE analysis of the DNA demethylation by ABH2 enzymes during an interaction with the dsDNA substrate containing m^1^A (**a**) or m^3^C (**b**). A comparison of the efficacy of the demethylation of damaged DNA substrates by ABH2 enzymes during interaction with m^1^A-containing or m^3^C-containing DNA (**c**). [Enzyme] = [DNA] = 0.5 µM. T = 37 °C, reaction time = 30 min. S is a substrate, P is a product of the cleavage of the demethylated-DNA chain by the restriction enzyme. Levels of accumulation of the product are presented as the average of three experimental values ± SD. * the data for ABH2 WT were obtained earlier [25] and are demonstrated here for comparison.

To establish the overall effect of the studied mutations on the activity of the ABH2 enzyme, the efficiency of the active demethylation of damaged dsDNA substrates by its mutant forms featuring V99A, R110A, F124A, S125A, R172A, and E175A amino acid substitutions was measured by a restriction-coupled assay followed by gel electrophoresis (Figure 8a,b). In total, 0.5 µM of the enzyme was preincubated with Fe^2+^ ions in the presence of αKG at 37 °C as well as 0.5 µM of the methylated 17 nt double-stranded substrate, containing ether m^1^A or m^3^C. After mixing, the reaction was stopped within 30 min, and the products were digested with a specific restriction endonuclease BstMB I to cleave the products of the active demethylation. The substrate and the shortened reaction product were then resolved in a gel, and thus, the activity of each enzyme on both substrates, m^1^A and m^3^C dsDNA, was determined.

As shown in Figure 6c, among all the enzymes studied, only the R172A mutant form retained the ABH2 WT level of activity toward m^1^A-containing dsDNA, more or less. As for the activity toward the m^3^C-containing substrate, it decreased from 75 to 55% for the ABH2 R172A enzyme compared to the WT. Looking ahead, it is fair to say that all of the substitutions in the current study led to the change in the preference between m^1^A and m^3^C shown for the ABH2 WT enzyme in our previous study [25]. The substitution of the Arg110 amino acid leads to a significant decrease in the enzymatic activity of the ABH2 enzyme toward m^1^A- and m^3^C-containing dsDNA—the product accumulation level drops to 20% (Figure 8c). Thus, it can be concluded that the substitutions of Arg110 and Arg172 both weaken the activity of the ABH2 enzyme. As for the V99A, F124A, S125A, and E175A mutant forms, the exhibited amounts of the cleaved product were about 10% in the case of the m^1^A-containing DNA and only 5% for m^3^C-containing DNA. Taking into account the multistage character of the detection method and the possible error it can create, it would be fair to say that the substitution of Val99, Phe124, Ser125, or Glu175 to alanine leads to a total loss of the ABH2 activity toward m^1^A and m^3^C. It is worth noting that three of four ABH2 mutant forms, comprising substitutions of these amino acids (except for E175A), demonstrated an increase in T_m_ values compared to ABH2 WT (Table 2). This may indicate that in the case of these mutant forms, we may be talking not only about increased thermal stability but also about an increase in the rigidity of the overall protein structure, or its regions, which subsequently complicate the conformational adjustments necessary for the catalytic state formation, making these enzymes inactive.

**Figure 9:**
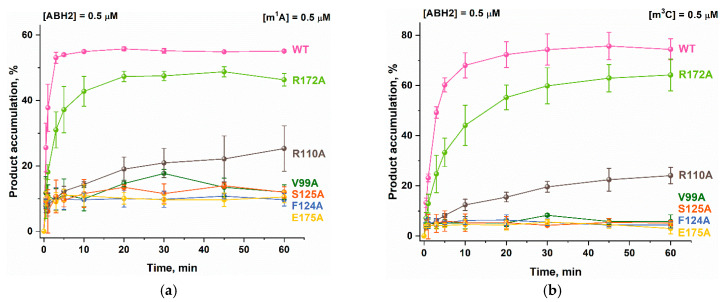
Active demethylation of m^1^A-containing (**a**) or m^3^C-containing (**b**) DNA substrates by ABH2 mutant forms V99A, R110A, F124A, S125A, R172A, and E175A. [Enzyme] = [DNA] = 0.5 µM. T = 37 °C. The levels of accumulation of the product at each time point are presented as the average of three experimental values ± SD. The data for ABH2 WT were obtained earlier [25] and are demonstrated here for comparison.

To clarify the details of the impact of the studied amino acids on the specific features of the reaction of the ABH2 enzyme with the methylated DNA substrates, the kinetics of product accumulation over time during the active demethylation of dsDNA containing m^1^A or m^3^C by ABH2 mutant forms featuring substitutions of V99A, R110A, F124A, S125A, R172A, or E175A were studied. Time courses of the substrate demethylation were obtained in a restriction-coupled assay of the ABH2 enzyme activity toward m^1^A- or m^3^C-containing 17 nt dsDNA substrates, with the visualization of the reaction products in PAAG (Figure 9); the time courses characterizing the process of the active demethylation of the m^1^A- or m^3^C-containing dsDNA by WT ABH2 were obtained in our previous work [25] and are presented here for the sake of comparison. It can be seen that the levels of overall product accumulation as well as the velocity of the process are lower for the ABH2 R172A mutant form compared to the WT enzyme, and the initial velocity of the process of the active demethylation of m^3^C-containing dsDNA seems to be reduced more markedly (Figure 9b). The substitution of Arg110 leads to the significant deceleration of the enzyme and the substantial loss of the efficiency of the active demethylation of the substrate containing m^1^A or m^3^C as a result.

**Figure 10:**
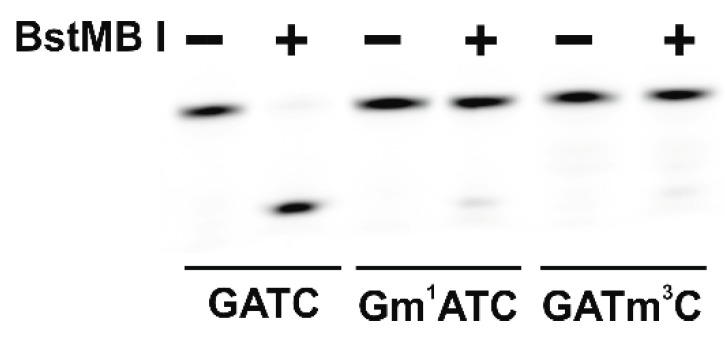
PAGE analysis of the cleavage of the DNA substrates containing m^1^A, m^3^C, or no methylated nucleotides (GATCs) by the restriction enzyme BstMB I, which is sensitive to the methylation of the GATC region.

The time courses of the product accumulation of the active demethylation of the m^1^A-containing DNA substrate by ABH2 mutant forms, containing V99A, F124A, S125A, or E175A substitutions, reach their maximum level (about 10%) during the first minutes of the reaction (Figure 9a). The same is true for the reaction with the m^3^C-containing DNA except that the maximum level of the product accumulation is about 5% (Figure 9b). This observation could be explained by the fact that the trace levels of the nonspecific cleavage of the methylated DNA by the restriction enzyme may play a prevailing role in the overall amount of the observed reaction product. We have tested the nonspecific restriction activity of the BstMB I enzyme in the reaction conditions used in this work for the mediated detection of the products of the active demethylation by ABH2 enzymes. As expected, we obtained the results of 10.1 ± 0.7% of the cleavage for the m^1^A-containing DNA and 6 ± 1%-for the m^3^C (Figure 10).

This result is in good agreement with the data obtained by Chen et al. [26], where the mutations of the Phe124 or Glu175 led to a complete loss of ABH2 repair activity toward the m^1^A-containing dsDNA substrate. At the same time, our result on the complete loss of activity of the V99A mutant form does not quite correlate with the data obtained by Monsen et al., since the authors demonstrated a significant decrease in the activity of this mutant form in relation to the m^3^C-containing DNA substrate but not the complete deactivation of the enzyme [28]. It is interesting that another mutant form studied by Chen et al. [26] and in a current work—R110A—did not exhibit any significant activity toward the m^1^A-containing dsDNA substrate according to the authors of the paper mentioned above [26]. As for the ABH2 S125A, the authors did not detect the dramatic loss in the activity of this mutant form toward the dsDNA substrate, although they emphasized that Ser125 has to play an important role in the base flipping and the substrate stabilization. The observed differences in the results obtained previously and by our group could be explained by the differences in the reaction conditions, such as the reaction temperature (4 and 37 °C, respectively), the presence of the Mg^2+^ ions, or the length of the DNA substrate (49 nt and 17 nt, respectively). Different factors possibly have an effect of different significance on the ABH2 mutant forms possessing the mutations that destabilize the protein more or less.

**Figure 11:**
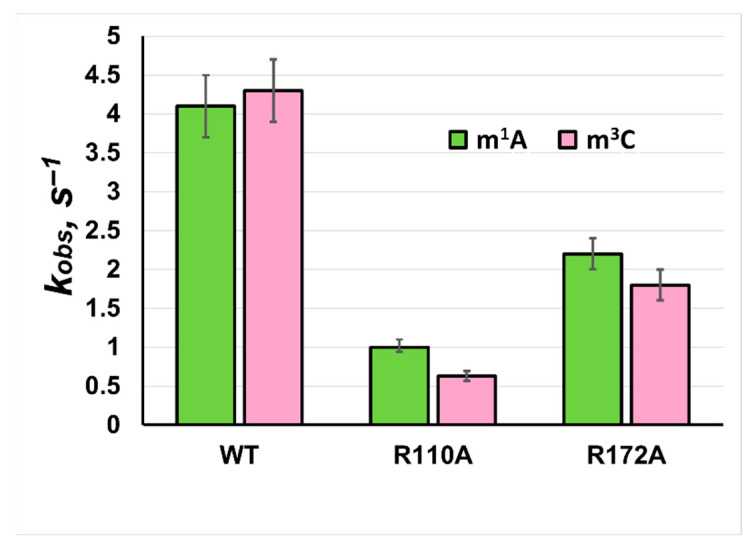
Observed rate constants *k_obs_* characterizing catalytic activity of ABH2 mutant forms R110A and R172A toward m^1^A- and m^3^C-containing DNA substrates. *k_obs_* values were determined by means of Equation (2). Error bars represent SD determined by mathematical data processing.

Observed rate constants *k_obs_* characterizing the process of the active demethylation of the m^1^A- or m^3^C-containing dsDNA substrates were determined with the help of Equation (2), which links this value with the obtained time courses and the EMSA data. The calculated *k_obs_* values, characterizing the catalytic repair of the methylated DNA substrates by ABH2 WT and its mutant forms R110A or R172A, are presented as a diagram in Figure 11 and are listed in Table 3. In the case of the ABH2 V99A, F124A, S125A, and E175A mutant forms, the *k_obs_* values are not presented due to the difficulties in the initial velocity evaluation. Kinetic analysis revealed that the substitution of Arg172 to alanine leads to a ~1.9–2.4-fold decline of the *k_obs_* values characterizing ABH2 catalytic activity toward m^1^A- and m^3^C-containing DNA substrates as compared to the WT enzyme (Table 3). The *k_obs_* value of the m^1^A demethylation reaction for ABH2 R110A was 4.1 times lower compared to the WT enzyme, and in the case of m^3^C, there was a 6.8-fold difference.

## 3. Materials and Methods

### 3.1. Oligodeoxyribonucleotides

The synthesis of the oligodeoxyribonucleotides (Table 4) was performed on an ASM-800DNA/RNA synthesizer (Biosset, Novosibirsk, Russia) by means of standard commercial phosphoramidites and CPG solid supports from Glen Research (Davis Dr, Sterling, VA, USA). The oligonucleotides were deprotected according to the manufacturer’s protocols and were purified by high-performance liquid chromatography. Oligonucleotide homogeneity was checked by denaturing 20% PAGE. Concentrations of oligonucleotides were calculated from their absorbance at 260 nm (A_260_). Oligonucleotide duplexes were prepared via annealing of oligonucleotide strands at a 1:1 molar ratio.

### 3.2. Site-Directed Mutagenesis

The following procedure was used to mutate the *abh2* gene at the sites of interest. Primers were synthesized as described above. Six mutant forms were designed by (1) substitution of a valine residue with alanine at position 99, (2) substitution of an arginine residue with alanine at position 110, (3) substitution of a phenylalanine residue with alanine at position 124, (4) substitution of a serine residue with alanine at position 125, (5) substitution of an arginine residue with alanine at position 172, or (6) substitution of a glutamate residue with alanine at position 175. The sequences of required forward primers were as follows (reverse primers were complementary to forward primers): 5′-GAGCACTGGCCAGAGCACAGGTATTCG-3′ (V99A), 5′-CACAGTGTGCCCGCAAAGCAGGCAACGTATGGCG-3′ (R110A), 5′-CTGACCTACACAGCTTCAGGCCTCACG-3′ (F124A), 5′-CCTACACATTTGCAGGCCTCACGCTG-3′ (S125A), 5′-CATCGGGGAGCACGCAGATGATGAAAGAG-3′ (R172A), and 5′-CCGAGATGATGCAAGAGAACTGGCCC-3′ (E175A). After PCR, methylated DNA was treated with the MalI restriction enzyme (SibEnzyme, Novosibirsk, Russia). These mixtures were transfected into *E. coli* strain ElectroMAX™ DH10B (Invitrogen, Waltham, MA, USA). Plasmid DNA was isolated from the resulting cell culture according to the protocol of the diaGene Isolation Kit (Novosibirsk, Russia). A NanoPhotometer (Implen, Munich, Germany) was used to measure the concentration of the isolated plasmid DNA, and the length of the DNA was determined in an agarose gel. Sequences of the transformed clones were confirmed by direct sequencing.

### 3.3. Enzyme Purification

**Figure 12:**
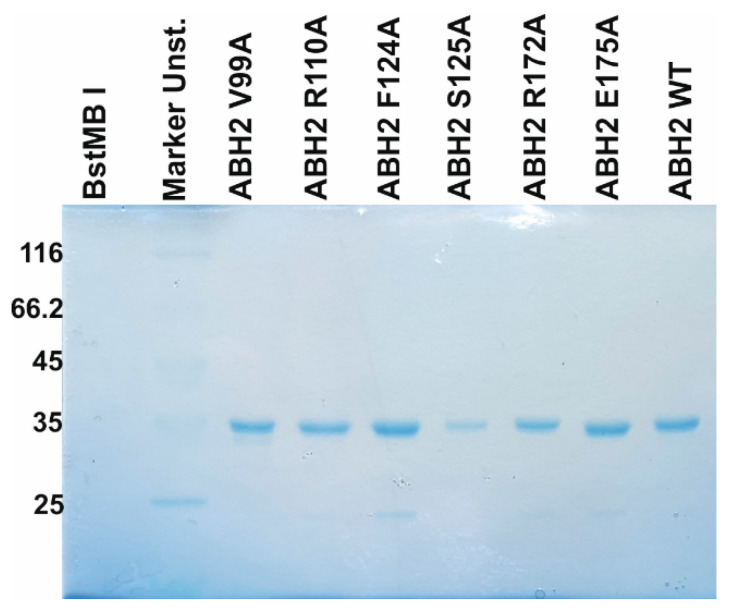
Analysis of protein purity of the V99A, R110A, F124A, S125A, R172A, and E175A ABH2 enzymes by SDS-PAGE. The proteins were stained with Coomassie Blue R-250 (Sangon Biotech, Shanghai, China).

To express the ABH2 mutant forms featuring substitutions of V99A, R110A, F124A, S125A, R172A, or E175A, *E. coli* ArcticExpress (DE3) cells (Invitrogen, Waltham, MA, USA) were transformed with plasmid pET28c encoding an N-terminal His_6_ tag and the corresponding gene. To purify the enzymes expressed as recombinant proteins, *E. coli* cells carrying one of the desired vector constructs were grown at 37 °C in 1 L of YT broth containing 50 µg/mL of kanamycin, 20 µg/mL of gentamycin, and 10 µg/mL of tetracycline until optical density at 600 nm (OD_600_) reached approximately 0.6. Then 0.2 mM isopropyl β-D-1-thiogalactopyranoside was added to induce the expression of the enzyme. Following a 24 h incubation at 16 °C, cells were harvested by centrifugation (5000× *g*, 20 min) and then resuspended in a buffer [20 mM HEPES-KOH (pH 7.8), 40 mM NaCl] with the addition of a mixture of EDTA-free protease inhibitors (Cocktail Protease Inhibitor, Wuhan Servicebio Technology, Wuhan, China), followed by cell lysis by means of the French press. All the purification procedures were carried out at 4 °C. The cell lysate was centrifuged at 40,000× *g* for 45 min. The supernatant was then passed through a 0.45 µm filter (Labfil, ALWSCI, Shaoxing, China), and the NaCl concentration in the supernatant was brought to 500 mM, whereupon the supernatant was mixed with Ni-sepharose (Ni IDA beads, Changzhou Smart-Lifesciences Biotechnology, Changzhou, China). The elution of the protein-containing fraction was carried out with a buffer composed of 500 mM NaCl, 20 mM HEPES-NaOH (pH 7.8), and 600 mM imidazole. Then, the NaCl concentration in the eluent was brought to 150 mM by dilution with 20 mM HEPES-NaOH (pH 7.8). The resulting solution was loaded on a 1 mL HiTrap-SP HP^TM^ column (Cytiva GE Healthcare Life Sciences, Marlborough, MA, USA). Bound protein was eluted with a linear 200→1200 mM gradient of NaCl. The homogeneity of the protein was verified by SDS-PAGE (Figure 12); the protein concentration was measured on the NanoPhotometer (Implen, Munich, Germany). Glycerol was added to the isolated fractions up to 50% (*v*/*v*). Purified proteins were stored at −70 °C before use.

### 3.4. CD Measurements

CD experiments were performed on a Jasco J-600 spectropolarimeter (Jacso Ltd., Tokyo, Japan) at 25 °C and a wavelength of 190 to 260 nm in quartz cells with a 0.1 mm light path length. The concentration of ABH2 mutant forms in the device cell was 20 µM. The experiments were conducted in a buffer consisting of 50 mM Tris-HCl with a pH of 8.0. The spectra were acquired at a bandwidth of 1.0 nm and a resolution of 1.0 nm, the registration speed was set to 50 nm/min, and the accumulation was set to 12. To describe the spectra, an online tool for the fitting and simulation of the CD spectra of proteins was used (https://bestsel.elte.hu, accessed on 22 April 2025) [35].

### 3.5. Fluorescence Thermal Shift Assay

The melting temperature was measured using the Quant Studio 5 Real-Time PCR System (Applied Biosystems, Waltham, MA, USA), in PCR tubes. Each tube contained 20 µL of solution containing 25 µM of protein in 50 mM Tris-HCl (pH 8.0) buffer and 5× ProteOrange dye (Lumiprobe, Westminster, MD, USA). The temperature was elevated steadily in 0.028 °C steps from 25.1 °C to 99.9 °C. The fluorescence of the ProteOrange dye was measured using excitation at 470 nm and emission at 558 nm. Measurements were taken in triplicate. The value of the melting temperature was calculated using the Boltzmann sigmoidal curve equation:

F = F_u_ + (F_b_ − F_u_)/[1 + exp(T_m_ − x/slope)]

where F is ProteOrange fluorescence emission, x is temperature, F_u_ is baseline fluorescence at low temperature, F_b_ is maximal fluorescence at the high temperatures, slope describes the steepness of the curve, and Tm is the melting temperature of the protein.

### 3.6. Gel Mobility Shift Assay for the DNA Substrate Binding by ABH2 Mutant Forms

The oligodeoxyribonucleotide substrates used in the EMSA are shown in Table 4. 6-carboxyfluorescein (FAM)-5′-labeled oligodeoxyribonucleotides containing m^1^A or m^3^C as damage were annealed to complementary oligodeoxyribonucleotides at 95 °C for 3 min in a buffer containing 50 mM Tris-HCl pH 8.0, 50 mM KCl, 10 mM MgCl_2_, 0.5 mM αKG, and 2 mM sodium ascorbate (EMSA buffer), then cooled to 22 °C. In parallel, one of the ABH2 mutant forms under study was diluted with EMSA buffer. The binding reactions were initiated by adding 5 μL of the DNA sample to 5 μL of the protein samples. Standard binding reactions contained 1.2 µM of FAM-labeled damaged DNA duplex with protein concentrations between 0.4 and 12.8 µM. The reaction mixtures were incubated for 10 min at 37 °C, then cooled for 5 min on ice. After the glycerol concentration in a sample was brought to 5%, the reaction mixtures were loaded on a nondenaturing 10% polyacrylamide gel (70:1 acrylamide:bis-acrylamide) in 0.5× TBE buffer [1× TBE buffer: 90 mM Tris, 90 mM boric acid, 2.4 mM EDTA]. After electrophoresis at 200 V in a cool box (at ~4–8 °C), the gels were visualized using a Molecular Imager^®^VersaDoc™ MP Imaging System (Bio-Rad, Hercules, CA, USA), and the bands were quantified by scanning densitometry in the Gel-Pro Analyzer software, v.4.0 (Media Cybernetics, Rockville, MD, USA). The fraction of bound DNA [F = DNA_bound_/(DNA_bound_ + DNA_unbound_)] was plotted against the protein concentration, and the binding data were fitted to the Hill equation (Equation (1)) with the OriginPro 8 software (OriginLab, Northampton, MA, USA). Titration series (nine points) were implemented in triplicate.

(1) $$F=F_{u}+\frac{F_{b}-F_{u}}{1+{\left(\frac{K_{d}}{E_{0}}\right)}^{h}}$$

where F is the fraction of bound DNA; F_u_ and F_b_ are the initial and final levels of F, respectively; h is the Hill coefficient; and *K_d_* is the effective dissociation constant of the complex.

### 3.7. Methylated DNA Repair Activity Assays

Analyses of the active demethylation of DNA substrates were carried out using FAM-5′-labeled oligodeoxynucleotides containing m^1^A or m^3^C as a lesion. The reactions were conducted in reaction buffer [50 mM Tris-HCl (pH 8.0), 50 mM KCl, 10.0 mM MgCl_2_, 1.0 mM αKG, 2.0 mM sodium ascorbate, and 40 μM (NH_4_)_2_Fe(SO_4_)_2_·6H_2_O] at 37 °C in a 10 µL reaction mixture. The dsDNA substrate and enzyme were separately preincubated for 2 min at the reaction temperature and combined at final concentrations of 0.5 µM each. The reaction was initiated by the addition of the enzyme. After 30 min, the reaction mixture was subjected to precipitation with a 2% solution of LiClO_4_ in acetone.

In the analysis of the kinetics of the active demethylation product accumulation, the reaction between m^1^A- or m^3^C-containing DNA substrates and the ABH2 mutant forms featuring substitutions of V99A, R110A, F124A, S125A, R172A, or E175A was stopped by means of precipitation at selected time points.

To visualize the product of DNA demethylation, each precipitated sample was treated with 2.5 U of restriction endonuclease BstMB I (SibEnzyme, Novosibirsk, Russia), which recognizes the GATC sequence. DNA methylation of this sequence blocks the restriction reaction maintained by BstMB I. The digestion of the ABH2 reaction product by BstMB I was carried out for 25 min at 37 °C. The reaction with BstMB I was quenched with 5 µL of a gel-loading dye containing 7 M urea and 50 mM EDTA. The oligodeoxynucleotides were resolved by electrophoresis in a 15% (*w*/*v*) polyacrylamide gel containing 7 M urea. PAGE was performed at 48 °C and a voltage of 200–300 V. The gels were visualized using the Molecular Imager VersaDoc™ MP Imaging System (Bio-Rad, Hercules, CA, USA), and the intensities of substrate and product bands were quantified by scanning densitometry in Gel-Pro Analyzer v.4.0 (Media Cybernetics, Rockville, MD, USA).

The percent of product was plotted as a function of time and the obtained data were analyzed via the following equation, under the assumption that an observed catalytic rate constant is a ratio of the initial velocity, *V*_0_, to the equilibrium concentration of the precatalytic complex [E·S]:

(2) $$k_{obs}=2V_{0}{\left(E_{0}+S_{0}+K_{d}-\sqrt{{\left(E_{0}+S_{0}+K_{d}\right)}^{2}-4E_{0}S_{0}}\right)}^{-1}$$

where *V*_0_ is the initial velocity estimated as the initial slope of the kinetic curve obtained under steady-state reaction conditions, *E*_0_ and *S*_0_ are the total concentrations of the enzyme and DNA, and *K_d_* is an equilibrium dissociation constant calculated in the EMSA by means of Equation (1).

### 3.8. MD Simulations

The crystal structures of ABH2 cross-linked to DNA containing modified nucleotide m^3^C (PDB ID: 3RZJ) or m^1^A (PDB ID: 3BUC) were chosen as initial structures [18,19]. Both structures include a lesioned nucleotide flipped into the active site of the enzyme, αKG as a cofactor, and the Mn^2+^ ion, which does not support catalysis while occupying the Fe^2+^-binding site [19]. To generate simulation structures, unresolved residues 204 to 206 from the loop region were added using Modeller 10.3 [36], disulfide cross-links necessary for crystallization were removed, DNA duplex sequences were adjusted to match the oligodeoxyribonucleotides used in the experiments, and Mn(II) was replaced with Fe(II).

Protonation states for ionizable amino acid side chains were assigned using the H++ server [37] and adjusted based on an inspection of the local hydrogen bond networks. System building and simulations were performed using the GROMACS 2022.6 MD package [38], with the AMBER 14SB-OL15 force field used to handle the protein and the substrate DNA [39,40,41,42]. The structures of ABH2-DNA complexes were solvated in a pre-equilibrated dodecahedral PBC box using the TIP3P water model and neutralized with 50 mM of KCl JC ions [43,44]. AMBER forcefield parameters for αKG, m^3^C, and m^1^A were generated by Antechamber, with RESP charges calculated by the REDD server [45,46,47,48,49]. Conversion to GROMACS-compatible formats was performed using the ACPYPE 2022.7.21 tool [50]. The Fe^2+^ active site ion was modeled as a nonbonded cationic dummy model [31].

A 1.0 nm cut-off was applied for nonbonded interactions, and the PME method was used to calculate long-range electrostatic interactions [51,52]. Hydrogen covalent bonds were constrained using the LINCS solver [53]. Initial energy minimization was performed using the steepest descent method. After that, each system was equilibrated under NVT and NPT ensembles sequentially for 1 ns using the Bussi thermostat and Parrinello–Rahman barostat [54,55], with solute heavy atoms position-restrained. Post-equilibration unrestrained MD simulations were run for 250 ns, and three replicas were made for each system, with one set for both m^3^C and m^1^A extended up to 500 ns. All three replicas were analyzed individually. Trajectory processing was performed using the integrated GROMACS toolset, with structural parameters analyzed using MDTRA 1.2 [56]. Images were generated in the open-source version of PyMOL Viewer 2.5.

## 4. Conclusions

A mutational analysis of Val99, Arg110, Phe124, Ser125, Arg172, and Glu175 residues, located in the area of the human DNA dioxygenase ABH2 active site, was performed by combining biochemical experimental approaches and MD calculations. MD simulations, performed in the current work and previously by our group, revealed the possible role of these amino acid residues in the catalytic process.

The substitution of Val99 leads to the changes in the positioning of L1 and L2 loops, participating in the fixation of the substrate at the ABH2 active site, as well as results in a suboptimal orientation of αKG due to the shortening of the distance between Gln112 and Asn159. These changes in the structure of the ABH2 active site result in a decrease in the binding affinity and catalytic activity of the V99A mutant form toward m^1^A- and m^3^C-containing dsDNA substrates, which was confirmed by the obtained experimental data.

The substitutions of Phe124 or Ser125 to alanine affected L2 loop positioning, and moreover, the modeling of the F124A mutant form led to frequent changes in the binding type of the Fe^2+^ ion and αKG, indicating the important role of this amino acid residue in the catalytic process carried out by ABH2. Both mutations in the L2 loop resulted in the complete loss of the catalytic activity of ABH2.

In the case of R110A, the loss of Arg110 side chain interaction with the DNA led to subsequent rearrangements in the hydrogen bond network at the active site of the enzyme, resulting in a significant decrease in the ABH2 R110A catalytic activity and a decline in a preference for the m^3^C-containing dsDNA substrate compared to the wild-type.

The E175A substitution led to the most noticeable changes in the ABH2 protein structure according to the CD analysis, and MD simulation indeed demonstrated significant disruption in the hydrogen bond network formed by residues Asp173, Glu175, Tyr122, Asn231, and Arg254 and the amino group of the everted methylated nucleobase, caused by this substitution. Predictably, the ABH2 E175A enzyme showed no catalytic activity to both studied DNA substrates, pointing out the extreme importance of this residue to the active demethylation carried out by ABH2.

Among all the enzymes studied, only the R172A mutant form retained the ABH2 WT level of activity toward m^1^A-containing dsDNA but demonstrated a decreased activity toward the m^3^C-containing substrate compared to the WT. MD simulations also showed that the replacement of Arg172 with Ala172 does not affect the stabilization of the damaged base in the active site or the coordination of αKG and Asp173 with the Fe^2+^ ion.

As a result, the data acquired clearly illustrate how most of the examined amino acid residues influence the enzymes’ catalytic activity, rather than their capacity to bind to DNA. Collectively, these findings provide insight into the molecular and kinetic implications of substituting active-site residues in the context of substrate recognition mechanisms.

## Figures and Tables

**Table 1:** Secondary structure content of ABH2 WT and its mutant forms according to the data of CD spectra fitting.

	α-Helix (%)	β-Sheet (%)	β-Turn (%)	Unordered (%)
**WT**	13 ± 3	34 ± 7	12 ± 2	42 ± 8
**V99A**	11 ± 2	34 ± 7	12 ± 2	43 ± 9
**R110A**	8 ± 2	36 ± 7	12 ± 2	44 ± 9
**F124A**	11 ± 2	33 ± 7	12 ± 2	44 ± 9
**S125A**	9 ± 2	36 ± 7	12 ± 2	43 ± 9
**R172A**	12 ± 2	35 ± 7	11 ± 2	42 ± 8
**E175A**	18 ± 4	28 ± 6	11 ± 2	42 ± 8

**Table 2:** The calculated T_m_ values for ABH2 variants.

	WT	V99A	R110A	F124A	S125A	R172A	E175A
**T_m_, °C**	46.7 ± 0.7	50.4 ± 0.4	49.1 ± 0.7	46 ± 1	49.1 ± 0.3	47.6 ± 0.2	47.7 ± 0.5

**Table 3:** A summary of kinetic parameters of DNA demethylation by ABH2 enzymes, as determined under steady-state conditions.

	*K_d_*, µM	*k_obs_*, s^−1^	
WT *	2.45 ± 0.07	4.1 ± 0.4	**m^1^A**
**V99A**	3.2 ± 0.3	-	
**R110A**	3.4 ± 0.2	1.0 ± 0.1	
**F124A**	3.1 ± 0.3	-	
**S125A**	2.7 ± 0.3	-	
**R172A**	2.10 ± 0.08	2.2 ± 0.2	
**E175A**	2.44 ± 0.06	-	
WT *	3.5 ± 0.1	4.3 ± 0.4	**m^3^C**
**V99A**	2.5 ± 0.6	-	
**R110A**	4.0 ± 0.3	0.63 ± 0.06	
**F124A**	4.4 ± 0.7	-	
**S125A**	4.0 ± 0.3	-	
**R172A**	2.6 ± 0.4	1.8 ± 0.2	
**E175A**	2.96 ± 0.04	-	

* The data for ABH2 WT were obtained earlier [25] and are demonstrated here for comparison.

**Table 4:** DNA substrates used in this study.

Shorthand	Sequence
m^1^A	5′-**FAM**-AGTTCAATG-**m^1^A**-TCTTCAT-3′ 3′-TCAAGTTAC T AGAAGTA-5′
m^3^C	5′-**FAM**-AGTTCAATGAT-**m^3^C**-TTCAT-3′ 3′-TCAAGTTACTA G AAGTA-5′
GATC	5′-**FAM**-AGTTCAATGATCTTCAT-3′ 3′-TCAAGTTACTAGAAGTA-5′

## Data Availability

Experimental data are available upon request to N.A.K. Tel. +7-383-363-5175, E-mail: nikita.kuznetsov@niboch.nsc.ru.
